# Speech recognition technology for assessing team debriefing communication and interaction patterns: An algorithmic toolkit for healthcare simulation educators

**DOI:** 10.1186/s41077-024-00315-1

**Published:** 2024-10-09

**Authors:** Robin Brutschi, Rui Wang, Michaela Kolbe, Kerrin Weiss, Quentin Lohmeyer, Mirko Meboldt

**Affiliations:** 1 D-MAVT, ETH Zurich, Leonhardstrasse, Zurich, 8092 Zurich Switzerland; 2 Simulation Center USZ, Universitätsspital Zürich, Huttenstrasse 46, Zurich, 8091 Zurich Switzerland

**Keywords:** Simulation, Healthcare, Debriefing, Education, Speaker diarization, Sociograms, Affective computing, Human-computer interaction

## Abstract

**Background:**

Debriefings are central to effective learning in simulation-based medical education. However, educators often face challenges when conducting debriefings, which are further compounded by the lack of empirically derived knowledge on optimal debriefing processes. The goal of this study was to explore the technical feasibility of audio-based speaker diarization for automatically, objectively, and reliably measuring debriefing interaction patterns among debriefers and participants. Additionally, it aimed to investigate the ability to automatically create statistical analyses and visualizations, such as sociograms, solely from the audio recordings of debriefings among debriefers and participants.

**Methods:**

We used a microphone to record the audio of debriefings conducted during simulation-based team training with third-year medical students. The debriefings were led by two healthcare simulation instructors. We processed the recorded audio file using speaker diarization machine learning algorithms and validated the results manually to showcase its accuracy. We selected two debriefings to compare the speaker diarization results between different sessions, aiming to demonstrate similarities and differences in interaction patterns.

**Results:**

Ten debriefings were analyzed, each lasting about 30 min. After data processing, the recorded data enabled speaker diarization, which in turn facilitated the automatic creation of visualized interaction patterns, such as sociograms. The findings and data visualizations demonstrated the technical feasibility of implementing audio-based visualizations of interaction patterns, with an average accuracy of 97.78%.We further analyzed two different debriefing cases to uncover similarities and differences between the sessions. By quantifying the response rate from participants, we were able to determine and quantify the level of interaction patterns triggered by instructors in each debriefing session. In one session, the debriefers triggered 28% of the feedback from students, while in the other session, this percentage increased to 36%.

**Conclusion:**

Our results indicate that speaker diarization technology can be applied accurately and automatically to provide visualizations of debriefing interactions. This application can be beneficial for the development of simulation educator faculty. These visualizations can support instructors in facilitating and assessing debriefing sessions, ultimately enhancing learning outcomes in simulation-based healthcare education.

**Supplementary Information:**

The online version contains supplementary material available at 10.1186/s41077-024-00315-1.

## Background

Debriefings are a core component of simulation training in healthcare [15, 22, 27, 28, 30, 33, 38]. Typically structured and guided by an instructor, they involve individuals or teams reflecting, analyzing, and discussing the actions and thought processes of the simulated case. The goal of debriefings is to learn from past experiences and to enhance future performance. Indeed, debriefings foster behavior change and team performance [15, 18, 30, 38]. Duvivier et al. [13] emphasize the debriefer’s role as a facilitator in guiding learners’ reflections for effective professional training. Their integrative models show that debriefing occurs in a dynamic, double-regulated context, requiring the trainer to continuously adapt and regulate their activity based on various components outlined in the Debriefing Simulation Trainer Activity Model (D-STAM). Yet, conducting debriefings is an art to be mastered [21], and debriefing approaches such as the Debriefing with Good Judgment, PEARLS [14], TeamGAINS [20], and the Diamond [17] provide important guidance for facilitation. Equally important, tools for assessing the debriefing quality or interactions such as the Debriefing Assessment for Simulation in Healthcare (DASH) [6], the Objective Structured Assessment of Debriefing (OSAD) [4, 38], and the Coding Scheme for Assessing Debriefing (DE-CODE) [38] help identifying characteristic debriefer-learner interaction patterns, for gaining insights into associations between debriefers’ communication and learners’ reflection and for comparing different debriefing approaches [36–39]. For example, lag sequential analyses (i.e., the method of assessing patterns “what tends to follow what?” in sequences) have shown that using “good judgement,” asking open-ended questions, paraphrasing, and storytelling can help learners reflect [24]. Social network analysis has identified four distinct debriefing interaction models [9]: *Line* (i.e., instructor interacting primarily with one learner), *Triangle* (i.e., instructor interacting primarily with two learners), *Fan* (i.e., individually with all participants), and *Star* (i.e., evenly among learners and instructors). A fifth *Net* pattern (i.e., strong interactions between all participants) [1] was associated with improved short-term individual and team learning [1]; a finding in line with research asserting that balanced interaction patterns are considered ideal for learning outcomes [7, 9].

Yet, getting to these results did so far require an intense data collection and analysis process, e.g., involving time-consuming, manual behavior coding, extensive data, and graphics production [3, 24, 41]. This manual process results in limitations in terms of efficiency, accurate assessment, and automated data analysis and visualization [35]. It also impedes the ability to uncover meaningful insights and patterns, emphasizing the need to develop systematic and automated debriefing assessment tools for efficient data utilization [2]. Sociograms are commonly used as a tool to visualize and analyze social relationships between individuals or groups, providing a means to interpret such interaction patterns.

Coggins et al. [8] highlighted the effectiveness of basic quantitative data measures, such as hand-drawn conversational diagrams and recorded timings of contributions, for providing immediate debriefer feedback in healthcare simulation settings [8]. These quantitative data measures collected during debriefings have the potential to greatly improve the debriefing process and enhance learning outcomes. We extend this research by exploring how a simple setup with a microphone and a PC and employing automatic speaker diarization technology may provide objective, non-biased, near real-time feedback on aspects of interaction patterns. We expect that

1. By employing a microphone and utilizing an algorithmic toolkit, which incorporates cutting-edge speaker diarization algorithms, (a) the speaker and (b) the duration of their speech during debriefing conversations can be accurately assessed automatically.
2. The identified speaker and speech duration data can be automatically transformed into visualisations, such as sociograms, representing debriefing communication and interaction patterns.

## Methods

### Study design and participants

We conducted this observational study at the Simulation Center of the University Hospital Zurich (Zurich, Switzerland). The study was conducted within a week-long teamwork simulation training in March 2022 for third-year medical students. The target group was chosen as an integrated part of the study curriculum of University Hospital of Zurich’s medical studies program for students to gain practical experience through simulation training. This is a follow-up study as part of the work performed by Weiss et al. [40], where the team explored the potential of mobile eye tracking and multi-person pose estimation to continuously collect data and measure teamwork during simulation-based training in healthcare. This study focuses purely on the debriefing sessions that took place after the simulation training. To elaborate, the study focused on the facilitator-guided post-event debriefing after a medical handover case simulation. Patient handover simulation cases involve healthcare providers practising effective transfer of patient care information. These simulations simulate various handover situations to improve communication skills, teamwork, and decision-making abilities, ultimately leading to better patient outcomes and reduced errors in real-world clinical settings. The inclusion criteria were third-year medical students and participants’ consent. Of the eligible 88 students, 64 actively participated in the simulation scenarios, while the remaining 24 students observed the scenarios and participated in the subsequent 16 debriefings [25, 40].

We conducted this study during the teamwork simulation focused on patient handover. Debriefings followed the “Debriefing with Good Judgment approach” [33, 34]. They were conducted in a circular setting, where two instructors (referred to interchangeably as debriefers in this paper) were positioned opposite the participants, forming a half-circle. All debriefings were led by the same two debriefers who were certified intensive care nurses with simulation-instructor training and more than 7 years of simulation and debriefing experience. Debriefings were conducted in (Swiss)-German, and due to the ongoing COVID-19 epidemic, participants were required to wear masks for safety. We identified the debriefing phases based on the Debriefing with Good Judgment approach (see Table 1). For each recording, we manually marked the start and end times of each stage. Additionally, we manually assigned the respective speaker identities at the beginning. The debriefing session is led by one of the debriefers who takes on the coordination role and initiates the session by starting with the introduction stage. This allows the debriefer to take the lead and provide important context, clarifying their role in guiding the debriefing session.

**Table 1:** Identification of debriefing agenda structure

Debriefing phase	Topics
$A_0$ - Introduction and setting the scene	Quick introduction from debriefer to what a debriefing session entails, including its goals, objectives, and agenda
$A_1$ - Reactions	Participants discuss their personal experiences during the simulation, sharing their thoughts and reflections
$A_2$ - “Facts” and analysis	Participants reflect on the communication during the handover and analyze aspects such as speed, clarity, and pronunciation. They provide specific examples of effective communication or areas where improvement is needed
$A_3$ - Conclusion	Participants provide specific examples of how the handover case can be improved
$A_4$ - Takeaways	Participants summarize their key learning from the simulation

### Study ethics

This study was granted exemption from the ethics committee of Canton Zurich, Switzerland (BASEC number: Req-2020-00200). No patients were involved, study participation was voluntary, and participants’ written informed consent was obtained.

### Data collection

The speech was recorded with an off-the-shelf room microphone ZOOM H2n audio recorder (Zoom Corporation, Tokyo, Japan). The audio recorder was positioned at the debriefing circle’s center. Prior to each debriefing session, we turned on the audio recorder to facilitate accurate data collection.

**Fig. 1:**
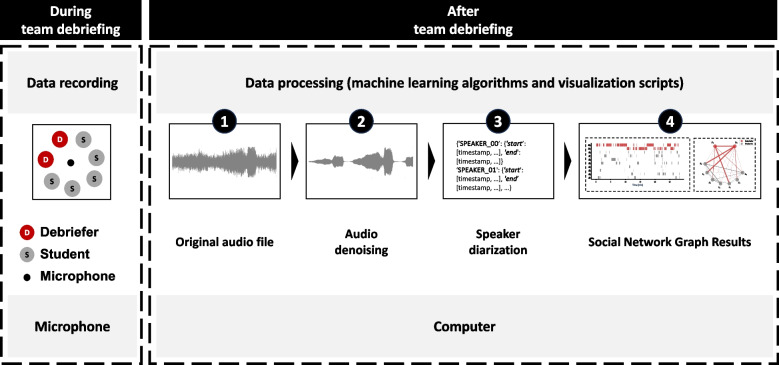
Audio data was recorded using an off-the-shelf audio recorder during debriefing sessions conducted in a circular setting. Machine learning algorithms were applied to de-noise the raw audio data and to identify and track the same speakers across the whole session. The tracked speaker sequence was further manually assigned with respective identity (debriefer/student), which was then utilized to generate interaction pattern graphs, including bar charts and sociograms. The generated pattern graphs visually depict the interactions between speakers in a way that facilitates easy analysis of the respective debriefing sessions

### Data processing

We processed the raw data through the use of machine learning algorithms for audio denoising and speaker diarization to analyze who spoke when to whom for how long (see Fig. 1).

#### Audio denoising

We trimmed the recorded audio files to correspond with the actual duration of the debriefings, ensuring that only relevant dialogue was included (i.e., the respective audio began with the start of the debriefing and ended precisely at the end of the debriefing). Subsequently, we performed denoising of the trimmed audio file using the open-source software FRCRN (Frequency Resolution Convolutional Recurrent Network) [11, 42]. FRCRN is a single-channel noise reduction method developed for enhancing speech in different noise environments to isolate crucial data and eliminate background noise. Audio denoising is the process of reducing unwanted background noise from an audio recording, which both enhances the performance of the speaker diariazation process and facilitates manual data inspection.

#### Speaker diarization

The goal of speaker diarization is to automatically determine who spoke when to whom. We subjected the denoised audio file to speaker diarization in order to identify individual speakers within the audio segments. We used PyAnnote [5, 29], a software package/library designed to automatically distinguish different speakers as well as their speaking sequence and speech duration. Using PyAnnote, we (a) transformed the voice signal into the frequency spectrum domain to extract distinct voice features, and (b) these voice features were then used to efficiently associate speakers’ identities via clustering algorithms. Utilizing this comprehensive list of data points―who spoke, when they spoke, and for how long―we were then able to automatically generate different visual representations of the debriefing process. To ensure accuracy and reliability, we conducted manual reconciliation checks to check our speaker diarization algorithms. We randomly selected 5 out of 10 recorded debriefing sessions and compared automated and manual speaker attributions.

### Data analysis and visualization

**Fig. 2:**
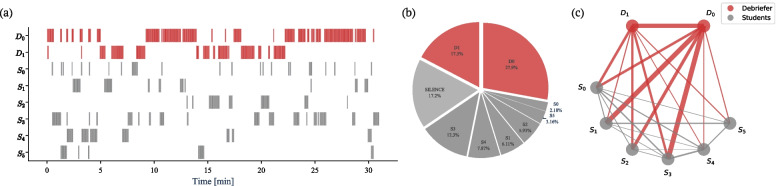
Overview of the algorithmic toolkit output. Conversation flow bar charts **a** visually depict speaking sequence and timing, highlighting active contributors with debriefers (*D*) in red and medical students (*S*) in grey. Speaking distribution pie chart **b** illustrates the proportional distribution of speaking time between debriefers, students, and silence. Sociogram network graph **c** analyzes interaction patterns and turn-taking dynamics between instructors and participants, showcasing relationships and communication flows with thicker lines indicating longer durations

The algorithmic toolset of speaker diarization creates a single event tuple for each speech segment (i.e., start time, end time, speaker). This setup allowed for different data analysis approaches: conversation flow bar charts (Fig. 2a), speaking distribution pie charts (Fig. 2b), and sociogram network graphs (Fig. 2c). We further highlight the Summary of Graphs, Objectives, and Usage suggestion in Table 2.

In the first step, we explain the algorithmic toolset. In a second step, we describe its application to two of the 10 debriefings (recording 02 and 05). These debriefings involved the same debriefers ($D_0$ and $D_1$), but different students. The purpose of this selective presentation is twofold: firstly to demonstrate how to read and interpret the output charts and secondly to identify similarities and differences between the two debriefings using the output charts as reference points.

#### Conversation flow bar charts

Conversation flow bar charts visually depict speaking sequence and timing, using bar charts to show the distribution of speaking segments (see Fig. 2a). Figure 2a displays individual speaking time, with debriefers (*D*) in red and medical students (*S*) in grey. Bars are arranged in descending order of speaking time, allowing quick identification of active contributors. This visualization highlights the activity level of participants and the sequence of speakers during the session. Our algorithmic toolkit determines the duration of each speaking segment for every person in a conversation by calculating the difference between their start and end times (see Eq. 1). This is performed individually for each person’s speaking segments. Mathematically, the duration of a speaking segment is obtained using the formula:

1 $$\begin{aligned} \text {Duration of Speaking Segment} = \text {End Time} - \text {Start Time} \end{aligned}$$

Once the duration of each speaking segment is determined, our code organizes the data by each person. However, it is important to note that the algorithm itself does not know the instructors involved in the simulation. Therefore, manual assignment of the instructors is required to accurately attribute the speaking segments to the respective individuals (see step 4 in Fig. 1). After the manual assignment, the code combines the durations of all the speaking segments for each person to calculate their total speaking time (see Eq. 2). Mathematically, the total speaking time for a person is computed by summing the durations of all their speaking segments, giving us the formula:

2 $$\begin{aligned} \text {Total Speaking Time} = \sum \text {Duration of Speaking Segments} \end{aligned}$$

The sorted events are analyzed to identify speaker transitions and generate graph edges. Each edge’s weight parameterizes the thickness and represents the duration of speech between the transitions, calculated by subtracting the start time of the previous event from the start time of the current event.

#### Speaking distribution pie charts

The speaking distribution pie visualizes the distribution of speaking time between debriefers and students (see Fig. 2b). Debriefers are represented in red, students in grey, and silence in light grey. The chart’s slices depict the proportional speaking time for each speaker or category. Mathematically, the algorithmic toolkit calculated the percentage of speech time for each person by dividing their duration of speaking segments by the total duration of the conversation, including pauses. This is represented by the Eq. 3:

3 $$\begin{aligned} \text {Percentage of Speaking Time} = \frac{\text {Duration of Speaking Segments}}{\text {Total Duration with Silences}} \times 100 \end{aligned}$$

#### Sociogram network graphs

Figure 2c displays the duration of speech segments, with thicker lines indicating longer durations. Sociograms can visualize communication direction using arrows to show flows from students to debriefers, debriefers to students, or students to students. The line connecting two nodes in a sociogram, representing a person speaking to another person, is commonly referred to as an edge. Our algorithmic toolkit automatically constructs a sociogram network graph by adding edges based on changes in speakers during the session. Each edge, denoted as (u, v), represents the transition from speaker u to speaker v. The weight of each edge, denoted as $w(u, v)$, corresponds to the duration of the speech segment between the speakers connected by the edge. Mathematically, the weight of an edge can be calculated as:

4 $$\begin{aligned} w(u, v)_ i = \text {{Start Time}}(v) - \text {{Start Time}}(u) \end{aligned}$$

Equation 4 captures the difference in start times between the current speech segment (speaker v) and the previous speech segment (speaker u). To calculate the total weight (sum of durations) of all the edges in the sociogram network graph, we used the Eq. 5:

5 $$\begin{aligned} W{(u,v)} = \sum \limits _{i} w(u,v)_i \end{aligned}$$

where *W*(*u*,*v*) indicates the total interaction time between speaker *u* and speaker *v*. It represents the summation of all individual edge weights, denoted as ($w_i$), where (i) ranges over all the edges in the graph. By summing up the weights of all the edges, we can obtain the total duration of speech segments captured in the sociogram. By incorporating these equations, the sociogram network graph provides a visual representation of the flow of communication and the relationships between speakers, highlighting the turn-taking dynamics in the session.

The sociogram could be adjusted accordingly to highlight certain information, such as focusing on students’ responses, which would be elaborated in the following sections.

**Table 2:** Summary of graphs, objectives, and usage suggestions of our algorithm toolkit output shown in Fig. 2

Graph	Description	Objective	Usage suggestions
**Conversation flow bar chart**	Shows who speaks when and for how long	To visualize the speaking times and participation of each participant during the session	Use this graph to overview the debriefing structure and each person’s engagement
**Speaking distribution pie chart**	Displays the distribution of speaking time among all instructors and students	To illustrate the overall distribution of speaking time between instructors and students, providing a clear picture of group dynamics	Employ this chart to quickly compare speaking time between different groups, ensuring balanced participation
**Sociogram network graphs**	Shows the relationships between participants, indicating direction, sequence, and frequency of interactions	To map out the communication patterns within the group, identifying who talks to whom, in what sequence, and how frequently	Utilize this graph to understand communication flow, detect key influencers or isolated members

### Algorithmic toolkit requirements

Our algorithmic toolkit requires a microphone for speaker diarization and mandates a computer to satisfy the minimum requirements of an operating system compatible with Python 3.6 or higher, a processor speed of 1 GHz or faster, a minimum of 4GB of RAM, and at least 10GB of free storage space, depending on the size of the dataset. Using our proposed system, the output charts can successfully be created within an expedited timeframe of 30 min.

## Results

### Debriefings

We applied our algorithmic toolkit to 10 recorded debriefings [40]. The average recording length was approximately 30 min. Five to ten students and two debriefers participated in each debriefing. Debriefing 02 and 05, which we chose to demonstrate the algorithmic toolkit, lasted approximately 27 min. The descriptives are shown in Table 3.

**Table 3:** Debriefing characteristics

Recording	Duration (min:s)	Students/debriefers
01	18:51	5/2
02	28:09	8/2
03	28:31	6/2
04	30:06	4/2
05	26:47	8/2
06	30:54	6/2
07	32:30	7/2
08	30:49	10/2
09	31:35	7/2
10	31:25	6/2

### Analysis

In the following analysis, we will highlight two different debriefing sessions, specifically recordings 02 and 05.

**Fig. 3:**
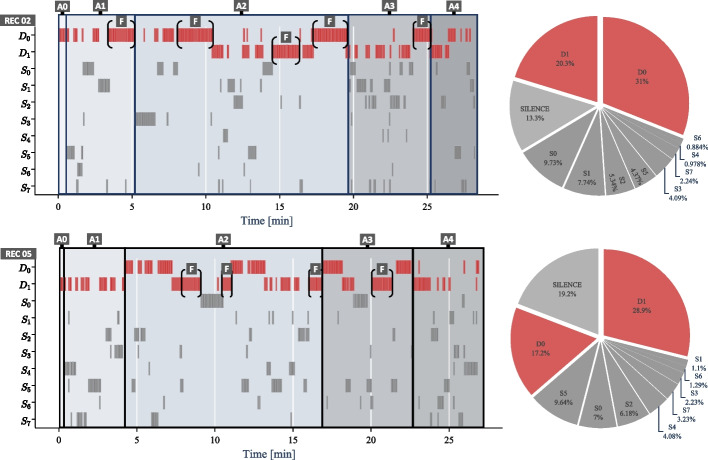
Manual semantic meaning overlay of debriefing stages of two speaker flow charts enables to perform stage-specific analysis. For example, in the discussion-centric stage *A*2, where students discuss and reflect on the hand-over simulation, recording 05 was dominated by student discussions whereas recording 02 showed longer feedback (*F*) sequences from debriefers. This suggests a more effective *A*2 stage in recording 05 in terms of students’ engagement

#### Conversation flow bar charts and speaking distribution pie chart

We present the manual semantically overlaid conversation flow bar charts (i.e., with the debriefing agenda stages ($A_0$ - $A_4$)), along with the speaking distribution pie charts, for the two indicated sessions in Fig. 3. The manual incorporation of semantic information allows for a more nuanced analysis, enabling us to examine the dynamic engagement of debriefers and students across different times in the debriefing. According to these visualizations, debriefing 02 and 05 differed with respect to conversation flow. While the speaking distribution pie charts indicate that during both debriefings, debriefers spoke longer than the students and all remained silent in 13–19% of the debriefing time, the conversation flow charts illuminate the difference: debriefers spoke more often than students in both debriefings, yet in debriefing 05, participants spoke more often than in debriefing 02 (see Fig. 3). More specifically, during debriefing 02, debriefer $D_0$ started and ended the debriefing ($A_0$, $A_1$, $A_4$) and actively contributed to maintaining the flow of the discussion―probably assuming the main debriefing role. Debriefer $D_1$, most likely assuming the role as co-debriefer, primarily participated during the analysis and conclusion ($A_2$ and $A_3$) to share observations. This dynamic is reversed in recording 05. In both debriefing sessions, the flow bar charts and speaking distribution pie charts revealed a pattern of alternating speech between $D_0$ and $D_1$, indicating a back-and-forth exchange of speaking turns among the debriefers, creating a varied conversation.

By visually comparing the two sessions, specific team interaction and engagement patterns could be identified. In each of the agenda stages, except during the introduction $A_0$, an “open-ended” question and answer (Q&A) format was observed. This format aims to engage participants by encouraging them to share their inputs and allows for diverse responses, fostering engagement, collaboration, and the exploration of different perspectives. However, within the agenda stages $A_1$ - $A_3$ of recording 02, it was noted that when none of the students provided answers to a question posed by one of the debriefers, the instructors chose to provide additional feedback (*F*) or speak further (see recording 02: *F*). This behavior is commonly employed to maintain the flow of the debriefing session and provide guidance or clarification to stimulate student thinking and encourage them to share their own thoughts or perspectives, as confirmed by manual validation through re-watching the actual debriefing recording. In contrast, recording 05 showed fewer and shorter instances of this behavior (see recording 05: *F*), indicating a more interactive and engaging communication flow of the student group. This observation was further supported not only by the conversation flow bar charts but also by the additional sociogram tool output charts.

**Fig. 4:**
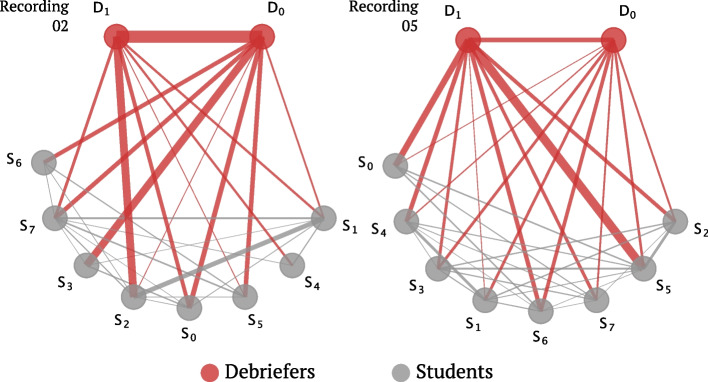
Sociogram analysis revealed similarities and differences between the two debriefing sessions. Strong student-student interactions were observed in both sessions, while debriefer-debriefer connections varied. Engagement levels differed between the sessions, with more interaction between debriefers and students in recording 05

#### Sociograms

The sociograms provide additional information on similarities and differences between the two debriefings (see Fig. 4). When examining the sociogram, one can focus on four main interaction directions: debriefer to debriefer, debriefer to student, student to debriefer, and student to student. In both debriefing sessions, there are strong interactions among the students themselves, for example, in recording 02 between students $S_1$ and $S_2$, and in recording 05 between students $S_3$ and $S_4$. Additionally, some students and debriefers are more active than others. In recording 02, there is a strong connection between the debriefers $D_0$ and $D_1$, which is not the case in recording 05. Regarding the interaction between debriefers and students, there is less engagement in recording 02 compared to recording 05. In recording 05, both debriefers interact with students, and there is a clear fan pattern where $D_1$ has longer interaction points with students compared to $D_0$.

There is substantial interaction among students, primarily due to the Q&A speech sequence discussed earlier, where one student’s statement is followed by another student’s statement. Regarding debriefer-to-student interactions, there are some students in a dominant role in both debriefing sessions (e.g., in recording 02 $S_0$ and $S_3$, and in recording 05 $S_0$, $S_5$, etc.), and there is not a significant difference in this aspect. Regarding student-to-debriefer interactions, recording 05 clearly shows a higher level of engagement, confirming the previous finding that students are taking a more active role, while debriefers are generally refraining from filling the silence with their own statements.

**Fig. 5:**
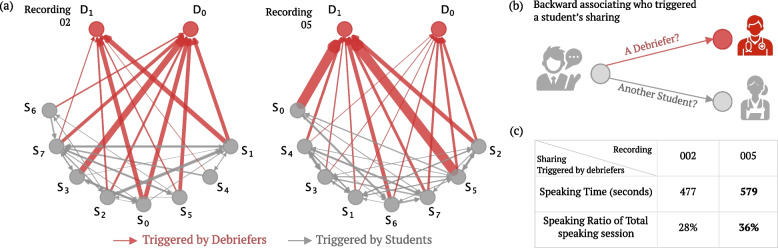
Backward association of students’ engagement in the discussion, focusing on what triggers their reflection and sharing. **a** shows the directional sociogram on backward-associating the students’ sharing for recording 02 and 05. The idea is further shown in **b**. **c** shows the numeric comparison between two indicated recordings. In recording 02, 28% of feedback from students was triggered by debriefers, while in recording 05, it increased to 36%. Hence, we can conclude that in recording 05, the debriefers were more successful in promoting feedback and facilitating student sharing

To further analyze the sessions in a student-centric manner, we further trace back what triggers the sharing of students and create a backward association fashion sociogram, shown in Fig. 5. For recording 05, both a higher student sharing rate triggered by debriefers and students could be observed from the figure. Focusing on the debriefers’ contribution, in recording 02, the debriefers triggered 28% of the feedback from students, whereas, in recording 05, this percentage increased to 36%. This suggests that in recording 05, the debriefers were more capable of prompting communication and sharing from the students, which may lead to a better learning experience for students.

### Data validation

To ensure accuracy and reliability, we conducted manual reconciliation checks to evaluate our speaker diarization algorithms. We randomly selected 5 out of 10 recorded debriefing sessions, evaluating the accuracy by comparing automated and manual attributions. The results of our manual reconciliation tests showed consistent accuracy across the recordings, with an average of 97.78% accuracy (see Table 4). We also manually explored defining the number of speakers but found it led to less accurate outcomes due to increased complexity. Short utterances lasting 0–10 s posed a challenge for speaker diarization systems like PyAnnote [5, 29]. Nevertheless, the accuracy provided by this method justifies its use.

**Table 4:** Data validation for speaker diarization

Recording	Time of interactions	Correct attribution	Accuracy
01	06:43	06:31	97.76%
03	08:18	08:06	97.59%
05	06:27	06:07	94.81%
08	04:12	04:00	95.24%
10	06:44	06:38	98.51%

## Discussion

This study explored the use of audio-based, minimally invasive speaker diarization technology to collect and process data to measure and visualize team debriefing interaction patterns in simulation-based training in healthcare. We expected that by employing a microphone and utilizing an algorithmic toolkit which incorporated speaker diarization algorithms, (a) the speaker and (b) the duration of their speech during debriefing conversations could be accurately assessed automatically, and that the identified speaker and speech duration data could be automatically analyzed and transformed into visualizations representing debriefing communication and interaction patterns. We found that the speech recognition technology reliably recorded and analyzed debriefing data. In what follows, we discuss the feasibility, contribution, and limitations of this study.

### Measuring team debriefing interaction patterns in simulation training

#### Identifying speaker and duration of speech

Using the algorithmic toolkit (see Fig. 1) allowed us to accurately identify who spoke when and for how long. This is an important advancement for both the study of team debriefings and debriefing faculty development. Our study enhances the simulation debriefing research community by providing applied technology that enables future researchers to more easily collect quantitative data on debriefing conversations. This addresses the current need to better understand effective debriefing practices due to limited empirical evidence [12, 32]. This aligns with previous research highlighting the effectiveness of basic quantitative data measures in advancing knowledge in the field [8, 19, 26]. Without much manual involvement, speech recognition technology may complement debriefing assessment by providing interaction data that used to require significant resources to collect. Our algorithmic toolkit did not require a large amount of human effort, such as rater training. Instead, it only required labeling the involved speakers accordingly. Our study achieved these results without the need for an intensive data collection and analysis process, such as time-consuming manual behavior coding or extensive data and graphics production, as seen in previous research by Kolbe et al. [24], Allen et al. [3], and Woolley et al. [41]. Our automatic process offers gains in terms of efficiency, accurate assessment, and automated data analysis and visualization. It also addresses the current need to develop systematic and automated debriefing assessment tools for efficient data utilization, as highlighted by Ali et al. [2]. This allows for the uncovering of meaningful insights and patterns.

#### Automatic analysis and visualisation

By applying our algorithmic toolkit, the automatically collected data could be further used to provide automatic analysis and visualizations in conversation flow bar charts, speaker distribution pie charts, and sociograms. To enhance the interpretation of results, we recommend manually adding the debriefing stages such as introduction $A_0$ and key topic 1 $A_1$ for better result interpretation. This does not take much time and does not demand high cognitive effort; in our case, it took us 30 min per debriefing session. Our tool’s output helps to limit human biases and aims to be objective and not prone to human errors. This capability of automated data visualization and quantifying interaction patterns, especially student responses, may be considered a significant advantage compared to traditional paper-pencil approaches. With this, we provide the community with a solution to the problem that obtaining such quantitative data typically requires experienced observers, whose training can be costly and time-intensive [16].We plan to make our audio-based social network analysis toolkit code available to the public. This is our first step towards enabling its use beyond research contexts. Additionally, we are actively collaborating with the Simulation Centre of the University Hospital Zurich to develop user-friendly software that can be easily used by non-technical staff. We welcome inquiries to the correspondence author and are open to sharing our insights and experiences with interested parties.

#### Pauses in debriefing and cultural background

The pauses during debriefing (labeled as silence) are shown in the pie diagram and flow chart. In our case, within a speak-up culture promoting freedom of expression and psychological safety [23], the percentage/occurrences of pauses across sessions do not show significant meaning. Through manual inspection of audio content, we found most pauses coincide with topic changes, turn-taking, processing or reflection, instructor emphasis, and for politeness. These findings align with Rochester’s studies on pauses over the past two decades [31]. However, in rare cases, we observed that debriefers sometimes interrupt participants or hastily fill silences, hindering reflection, potentially lead to missing learning opportunities. Moreover, research shows individuals may react quickly due to social expectations or discomfort with silence, which can disrupt effective communication [10]. Therefore, understanding the context of each individual pause is crucial for further analysis and could be a focus for future researches.

Effective debriefing sessions, as indicated by quality indicators linked to graphs such as speaker identification and duration of speech, include balanced speaking time among participants, active engagement from all involved, minimal prolonged silence, structured discussions using frameworks, clear communication flow as depicted in communication pattern graphs, and effective use of specific, actionable feedback. However, it is challenging to establish universal percentages for silence or speaking time that definitively signify effectiveness, as these metrics can vary significantly based on factors such as the complexity of topics discussed, cultural differences in communication norms, and the specific objectives of the debriefing session. Therefore, evaluating the quality of debriefings requires consideration of context-specific dynamics and goals to accurately assess engagement, communication effectiveness, and overall session impact.

### Limitations

While our research has provided valuable insights into the quantification of interaction among students and debriefers in simulation training debriefings, there are several limitations that need to be acknowledged.

Limited sample size: The study was conducted with a relatively small sample size of 10 debriefing sessions, each approximately 30 min long. This sample size might not fully capture the diversity and variability of interaction patterns that could be present in a larger and more extensive dataset.

Limited debriefer variability: Throughout the study, the same two instructors were involved in all 10 debriefing sessions, following the same agenda structure. This lack of instructor variability and agenda structure diversity may limit the generalizability of the findings. Different instructors may bring unique teaching styles, communication strategies, or approaches to facilitating interactions. Therefore, the results should be interpreted with the understanding that they may not fully account for the potential influence of different instructors on the observed interaction patterns. Future studies could involve a more diverse instructor pool to explore how different instructional styles impact the quantification and understanding of interaction in simulation training debriefing sessions.

Contextual limitations: The research was conducted at a single, academic, Western institution within a specific simulation training context (i.e., handover). This may limit the applicability of our findings to other simulation training settings. It is important to consider the contextual factors that may influence the patterns of interaction in different scenarios.

Technology constraints: Our proposed system relies on audio denoising and speaker diarization algorithms. While this approach allows for automatic analysis, factors such as ambient noise, device placement, and audio quality may affect the accuracy and reliability of the generated data. We observed a small chance of automatic attribution errors, especially for speech segments that are under 10 s long.

Human interpretation: Although our system provides visualizations of interaction patterns, the interpretation of these visualizations still requires human intervention, such as assigning the speaker labels to the participants’ names and making general judgments when interpreting the results. There is a possibility of subjective bias in our analysis, as different observers may interpret the data differently.

Lack of comparative analysis: Our study did not compare the effectiveness of our system against alternative methods of quantifying interaction in simulation training debriefing sessions.

Universal metrics and cultural differences: Establishing universal metrics for interpreting graphs in debriefing sessions is challenging due to factors like topic complexity, cultural communication norms, and session objectives. These variables influence how metrics such as speaking time and silence are interpreted, necessitating context-specific assessments to gauge session effectiveness and participant engagement accurately.

### Further research needs

Future research should consider comparing our proposed audio-based approach with other modalities or approaches to determine the strengths and limitations of different methodologies. These limitations should be taken into account when interpreting the findings of our research and should guide future studies in further exploring and refining the quantification of interaction in simulation training debriefing sessions.

## Conclusion

In conclusion, our research paper introduces a novel audio-based social network analysis generation toolkit to address the challenges of quantifying the interaction between students and instructors in simulation training debriefing sessions. By utilizing a microphone and running the audio recordings through a speaker diarization algorithm, our proposed methods enable almost real-time automatic feedback. This approach not only requires minimal effort and hardware but also provides visualizations of interaction patterns, including sociograms that are typically challenging to obtain through manual observation alone. The results obtained from our toolkit can have significant implications for improving training sessions and enhancing participant engagement. By analyzing the interaction data, instructors can gain valuable insights that can be used to optimize sessions and offer targeted feedback to enhance the learning experience. In summary, our proposed audio-based social network analysis generation toolkit offers a valuable tool for quantifying and visualizing interaction patterns in simulation training debriefing sessions. This research serves as a foundation for future advancements in the field and has the potential to significantly enhance the learning outcomes of simulation-based training programs and other contexts where communication and response rate are important for learning. Our novel algorithmic toolkit can serve as a valuable resource for debriefers, akin to a “debriefing for debriefers,” empowering them with insights and information to enhance their debriefing practices and improve the effectiveness of their sessions.

## Supplementary information

Supplementary Material 1.

## Data Availability

The datasets used and analyzed during the current study are available from the corresponding authors on reasonable request.

## Abbreviation

FRCRN: Frequency Resolution Convolutional Recurrent Network
