# Supplementary material for: Speech recognition technology for assessing team debriefing communication and interaction patterns: An algorithmic toolkit for healthcare simulation educators
Source: Adv Simul (Lond). 2024 Oct 9;9:42. doi: 10.1186/s41077-024-00315-1 (PMC11465542; doi:10.1186/s41077-024-00315-1)
Supplement: Supplementary file 1 — Supplementary Material 1. [file 41077_2024_315_MOESM1_ESM.pdf]

1 Appendix

1.1 Manual data validation for speaker diarization

1.1.1 Recording 01

Table 1 Manual Speaker Diarization Check for recording 01

Interaction	Start Time	End Time	Speaking Time	Speaker	Manual Check
1	00:00	00:29	00:28	S_1	correct
2	00:29	00:43	00:14	S_2	correct
3	00:48	00:54	00:06	S_1	correct
4	00:54	01:24	00:29	S_4	correct
5	01:25	01:42	00:17	S_1	correct
6	01:42	02:09	00:27	S_3	correct
7	02:14	02:47	00:32	S_5	correct
8	02:57	03:04	00:06	S_1	wrong speaker attribution
9	03:05	03:43	00:38	S_0	correct
10	03:43	04:47	01:03	S_1	correct
11	04:47	05:00	00:13	S_6	correct
12	05:00	05:14	00:14	S_3	correct
13	05:12	05:19	00:06	S_6	wrong speaker attribution
14	05:18	05:34	00:16	S_3	correct
15	05:36	05:49	00:13	S_6	correct
16	05:49	05:55	00:06	S_3	correct

1.1.2 Recording 03

Table 2 Manual Speaker Diarization Check for recording 03

Interaction	Start Time	End Time	Speaking Time	Speaker	Manual Check
1	15:06	15:14	00:07	S_3	wrong speaker attribution
2	15:17	17:23	02:05	S_7	correct
3	17:26	17:57	00:31	S_5	correct
4	17:57	18:29	00:31	S_7	correct
5	18:30	19:54	01:24	S_3	correct
6	19:59	20:30	00:30	S_4	correct
7	20:30	20:35	00:05	S_5	wrong speaker attribution
8	20:42	20:54	00:12	S_6	correct
9	20:54	21:59	01:04	S_7	correct
10	22:01	22:30	00:29	S_4	correct
11	22:30	23:36	01:05	S_7	correct

1.1.3 Recording 05

Table 3 Manual Speaker Diarization Check for recording 05

Interaction	Start Time	End Time	Speaking Time	Speaker	Manual Check
1	05:03	05:13	00:10	S_2	correct
2	05:13	05:27	00:14	S_6	correct
3	05:28	05:34	00:05	S_2	wrong speaker attribution
4	05:36	05:43	00:07	S_2	wrong speaker attribution
5	05:46	05:54	00:08	S_2	correct
6	05:54	06:17	00:22	S_8	correct
7	06:20	07:11	00:50	S_2	correct
8	07:13	07:44	00:31	S_3	correct
9	07:45	07:53	00:08	S_1	wrong speaker attribution
10	07:51	09:00	01:09	S_3	correct
11	09:04	10:27	01:23	S_5	correct

1.1.4 Recording 08

Table 4 Manual Speaker Diarization Check for recording 08

Interaction	Start Time	End Time	Speaking Time	Speaker	Manual Check
1	14:03	14:09	00:05	S_9	wrong speaker attribution
2	14:06	14:21	00:14	S_6	correct
3	14:23	15:07	00:44	S_1	correct
4	15:11	15:30	00:18	S_1	correct
5	15:31	16:02	00:31	S_5	correct
6	16:02	17:24	01:21	S_1	correct
7	17:27	17:52	00:24	S_1	correct
8	17:54	18:10	00:16	S_1	correct
9	18:10	18:18	00:07	S_8	wrong speaker attribution
10	18:19	18:36	00:16	S_1	correct
11	18:36	19:17	00:40	S_8	correct

1.1.5 Recording 10

Table 5 Manual Speaker Diarization Check for recording 10

Interaction	Start Time	End Time	Speaking Time	Speaker	Manual Check
1	20:45	21:08	00:22	S_5	correct
2	21:08	21:44	00:35	S_1	correct
3	21:44	22:52	01:08	S_3	correct
4	22:54	24:33	01:39	S_3	correct
5	24:36	24:43	00:06	S_1	wrong speaker attribution
6	24:43	24:57	00:14	S_3	correct
7	25:01	25:16	00:15	S_3	correct
8	25:17	25:26	00:08	S_5	correct
9	25:26	26:10	00:43	S_3	correct
10	26:12	26:36	00:24	S_3	correct
11	26:40	27:11	00:30	S_1	correct

1.2 Automatic generated graph results for measuring team debriefing interaction patterns

1.2.1 Example of a dynamic sociogram

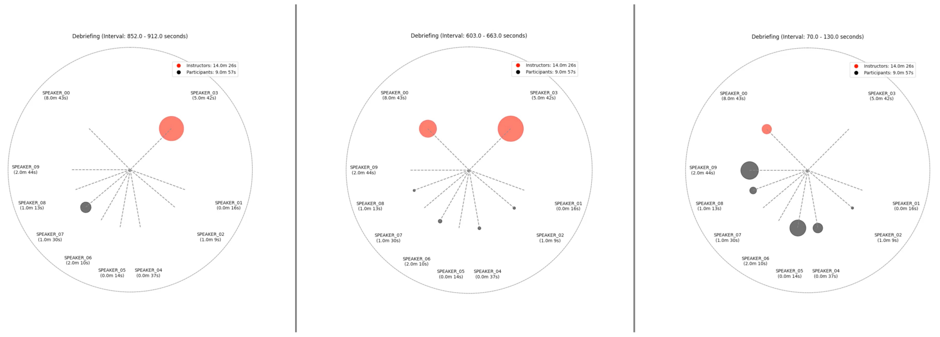

Fig. 1 A dynamic sociogram that visualizes the interaction patterns of speakers over time. The plot represents speakers as bubbles, with the size indicating the duration of their speaking time and the color representing their role. The animation shows how these interaction patterns evolve throughout the conversation.

1.2.2 Example of an information table

Table 6 Summary table summarizing the speech duration and the overall distribution in percentage of each speaker

SPEAKER	Duration in seconds (min:sec)	Percentage of speech time
S_0	523.99 seconds (08:43)	31.02%
S_3	342.51 seconds (05:42)	20.28%
S_9	164.33 seconds (02:44)	9.73%
S_6	130.80 seconds (02:10)	7.74%
S_7	90.20 seconds (01:30)	5.34%
S_8	73.79 seconds (01:13)	4.37%
S_2	69.08 seconds (01:09)	4.09%
S_4	37.78 seconds (00:37)	2.24%
S_1	16.52 seconds (00:16)	0.98%
S_5	14.93 seconds (00:14)	0.88%
SILENCE / PAUSE	225.01 seconds (03:45)	13.32%
